# Effect of Online Psychological Intervention on Burnout in Medical Residents From Different Majors: An Exploratory Study

**DOI:** 10.3389/fpsyg.2021.632134

**Published:** 2021-05-07

**Authors:** Jian Wang, Bijia Song, Yun Shao, Junchao Zhu

**Affiliations:** ^1^Department of Anesthesiology, Shengjing Hospital of China Medical University, Shenyang, China; ^2^Department of Anesthesiology, Beijing Friendship Hospital of Capital Medical University, Beijing, China; ^3^Department of Psychiatry, Shengjing Hospital of China Medical University, Shenyang, China

**Keywords:** medical residents, depression, anxiety, stress, burnout, psychological intervention

## Abstract

**Background:** Work-related stress among healthcare professionals poses a serious economic and healthcare burden. This study aimed to investigate the prevalence of burnout as well as anxiety, depression, and stress in medical residents from different majors, and assess the effects of an online psychological intervention on the mental health status of medical residents with a high degree of burnout.

**Methods:** We conducted an online survey that collected information on the demographics, mental health, and burnout conditions of medical residents from Shengjing Hospital. The mental health condition was assessed by the Depression, Anxiety, and Stress Scale (DASS)−21. Further, burnout was assessed by the Maslach Burnout Inventory (MBI). Medical residents with a total MBI score between 50 and 75 were selected to receive online psychological intervention for 3 months.

**Results:** Two-hundred and ten medical residents completed the questionnaire, of whom, 63 residents with an MBI score between 50 and 75 received the 3-month online psychological intervention. Anesthesia residents showed the highest level of depression, anxiety, and stress, and presented with a lower sense of personal accomplishment, higher emotional exhaustion, and higher depersonalization. Furthermore, pediatric residents had the second highest DASS and MBI scores following anesthesia residents. Following the online psychological intervention, negative emotional states and burnout levels were significantly lower among anesthesia and pediatric residents. There were no differences in the level of stress and sense of personal accomplishment pre- and post-online psychological intervention among the different majors.

**Conclusion:** Our findings revealed high levels of burnout, as well as depression, anxiety, and stress symptoms in medical residents, with marked differences among different majors. The online psychological intervention effectively improved emotional exhaustion, and depersonalization, and relieved the psychological problems such as anxiety and depression in medical residents.

## Introduction

Burnout is a symptom of emotional exhaustion most likely to occur in the healthcare industry. The World Health Organization (WHO) defined burnout as an occupational phenomenon in May 2019. Maslach and Jack first described this condition, which is characterized by emotional exhaustion, feelings of low personal accomplishment, and depersonalization (Maslach and Jackson, 1986). Gabbe et al. (2002) also noted that emotional exhaustion may be the most important component of burnout, due to an excessive workload, along with loss of autonomy and control of the work environment. Previous data showed that work-related stress among healthcare professionals has become a serious health problem for workers and the world economy. The syndrome has reached epidemic levels among both doctors in practice and in training, with a prevalence near to or exceeding 50% (Rodrigues et al., 2018). During their medical residency, resident physicians must develop specific skills in their chosen area in order to maintain the quality of patient care (Zis et al., 2014). During this period, they are subjected to sleep deprivation, a high workload, and unsatisfactory salaries, as well as taking on many responsibilities. This combination of factors makes them vulnerable to developing burnout, thus interfering with the individual's ability to handle diagnostic dilemmas, establish rapport, and work through complex treatment decision-making (Thomas, 2004). Bianchi's et al. studies proved that burnout overlaps with depression (Bianchi et al., 2015, 2020). Thus, it is necessary to develop guidelines for active coping and social support, to help medical residents maintain their well-being, both at the workplace and at home. Assessing the potential effectiveness of psychological intervention within this population is also crucial. Although effective treatments are available, it was reported that two-thirds of people with mental disorders did not seek help due to the stigma in seeking mental health services (Hankir et al., 2014). Internet-based interventions provide an alternative to face-to-face therapy, improving mental health in people who may not seek help due to stigma or other reasons. Internet-based interventions are anonymous, self-paced, and easily accessible. The high scalability and penetration of Internet-based interventions also offers advantages over face-to-face treatment (Mak et al., 2017). A previous study found that mindfulness decompression intervention and mindfulness-based cognitive therapy are the most widely applied treatment, are efficacious for the treatment of depression and anxiety, and can improve the level of burnout in clinical and non-clinical populations (Mak et al., 2017). However, the efficacy of Internet-based interventions on mental health conditions and burnout in medical students has not been assessed.The aim of our study was to determine the burnout rate and negative emotional states among medical residents of different specialties, and to assess the effect of online psychological intervention on these aspects in medical residents with a high degree of burnout.

## Methods

### Participants

The study was completed from 2019, 12.16 to 2020, 8.31 among year 1, 2, and 3 medical residents who worked in Shengjing Hospital of China Medical University. Ethical approval for this study was granted by the Human Research Ethical Committee of Shengjing Hospital of China Medical University (2017PS20K). In order to ensure privacy of the research subjects and ensure that the information obtained was true and credible, personal information was not disclosed to any person or organization when recruiting the research subjects. And we all got informed consent from the participants recruited.

### Procedures

Two online questionnaires that were designed and published on the Chinese Star Survey website were used. The first online questionnaires were answered before online psychological intervention. These contained questions on demographics; work conditions, including communication time during working, working environment and number of night shifts per month; health conditions, which was divided into healthy, average, and poor; and burnout and mental health conditions using the Maslach Burnout Inventory (MBI) and the Depression Anxiety Stress Scale (DASS-21), respectively. According to results of the first online questionnaires, medical residents with a total MBI score between 50 and 75 were selected to receive an online psychological intervention for 3 months. After the online psychological intervention, they were required to complete the second online questionnaires. These contained questions on 22 items of the Maslach Burnout Inventory (MBI) and 21 items of the Depression Anxiety Stress Scale (DASS-21). In order to ensure the research subject's privacy and ensure that the information obtained is true and credible, when we recruited research subjects, we promised not to disclose the personal information to any person or organization. Second, because medical students were recruited, they had a certain understanding of psychological problems; thus, the degree of stigma will be lower. Finally, our experts are trained and qualified professionals in psychology who were capable of reducing the stigma by providing psychological education. Three psychological experts from our team discussed with the department leaders and designed an online psychological intervention scheme. The components of the online psychological intervention included:

(1) Mindfulness decompression intervention: medical residents with a total MBI score between 50 and 75 participated in an 8-week 30–45 min abridged mindfulness decompression intervention. In the program, videos of stretching and audios instructions for performing a body scan and sitting meditation were provided to the participants to guide them through their exercises.

Psychoeducation: The counselors helped medical residents increase their self-confidence, identify their potential talents, and relieve mental stress through QQ, WeChat. At length, an anonymous communication platform was provided under the guidance of our professional psychologists once a week. Each psychological education duration was 1–3 h. In this type of network communication, the individual's problems were identified, and targeted psychotherapy was then provided.

Because of the epidemic, many online communication software, such as QQ, Tiktok and wechat, are becoming more and more perfectin China. More and more people relieve pressure through this form of network communication.We use this popular model to provide an anonymous communication platform under the guidance of our professional psychologists, so as to achieve the purpose of relieving stress.

### Sample Characteristics

**Figure 1:**
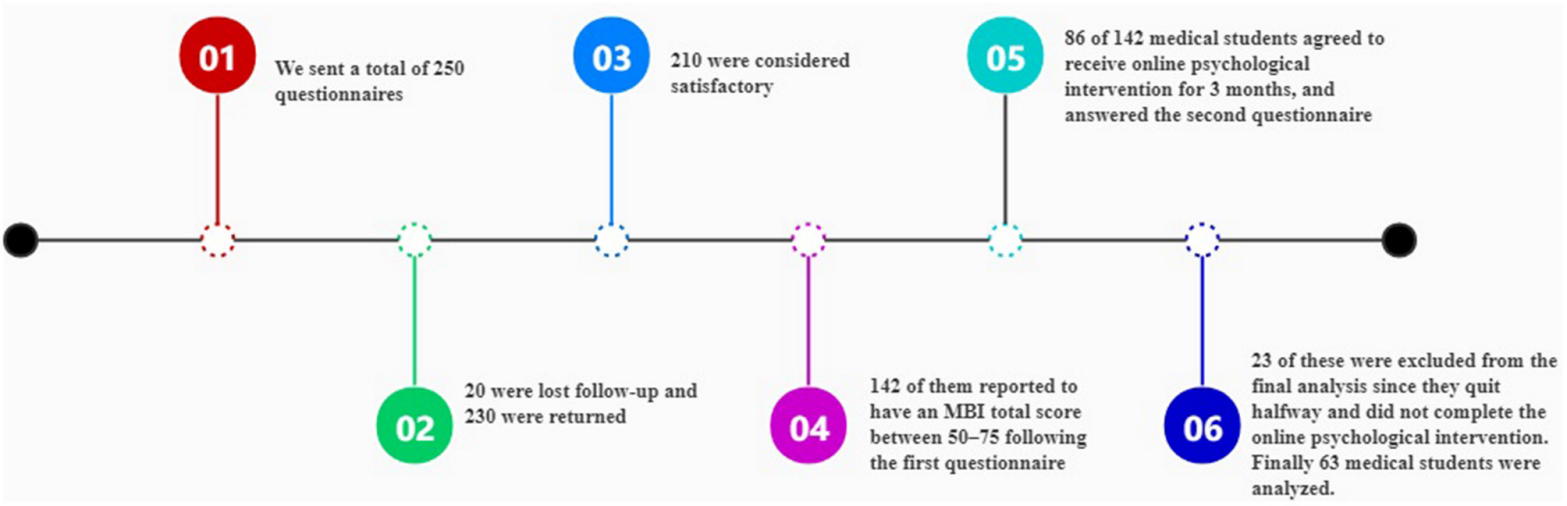
Timeline of the study.

We sent a total of 250 questionnaires; 230 were returned and 210 were considered satisfactory. Of these 210 participants, 142 of them reported to have an MBI total score between 50 and 75 following the first questionnaire (28 males and 114 females). While 56 refused, 86 of them agreed to receive online psychological intervention for 3 months and answered the second questionnaire, to perform a comparative analysis with the first questionnaire. However, 23 of them were excluded from the final analysis since they quit halfway and did not complete the online psychological intervention. Finally, 63 participants were included in the comparative analysis before and after online psychological intervention (19 males and 44 females) (Figure 1).

### Materials

#### Depression, Anxiety, and Stress (DASS-21)

This 21-item short scale allows for the simultaneous assessment of the three emotional states of depression, anxiety, and stress, is easy to apply in both clinical and non-clinical settings, and is suitable for use in different age groups in China, including medical students (Gong et al., 2010). Each item has a four-point Likert scale. The rating choices are “never applied to oneself” (0 points), “some degree/some of the time” (1 point), “considerable degree/a good part of time” (2 points), and “very much/ most of the time” (3 points). For depression, the criteria were normal (0–9 points), mild (10–13 points), moderate (14–20 points), severe (21–27 points), and extremely severe (28+ points). For anxiety, the criteria were normal (0–7 points), mild (8–9 points), moderate (10–14 points), severe (15–19 points), and extremely severe (20+ points). For stress, the criteria were normal (0–14 points), mild (15–18 points), moderate (19–25 points), severe (26–33 points), and extremely severe (34+ points). Each subscale's score is the doubled sum of the seven items (Osman et al., 2012).

#### Maslach Burnout Inventory (MBI)

The MBI**-**Human Services version is considered a reliable and valid tool to evaluate burnout state related to occupational stress among the Chinese population (Maslach Burnout Inventory, 2015). The MBI**-**Human Services version consists of 22 items with three subscales: emotional exhaustion (EE, 9 items), depersonalization (DP, 5 items), and personal accomplishment (PA, 8 items). Responses are provided using a 7-point Likert-type scale with response options ranging from 1 (never) to 7 (daily). EE reflects “feelings of being emotionally overextended,” DP reflects an “impersonal response toward recipients of one's service,” and PA reflects “feelings of competence and successful achievement in one's work with people,” which indicates the opposite of a burnout state. Higher levels of burnout state are associated with higher EE and DP scores and lower PA scores (Maslach Burnout Inventory, 2015). A score ≥ 27 on the EE subscale score and/or ≥13 on the DP was considered as moderate to high-level burnout. The PA measures feelings toward one's own success and competence in work. This subscale has an inverse relationship with burnout (low ≥ 39, average 38–32, high ≤ 31). A total score of 50 or less indicates a good working condition, a total score of 50–75 indicates that there is a certain degree of job burnout, and psychological adjustment is required. Individuals with a total score of 75–100 are recommended to take a vacation and leave the job for adjustment. Individuals with a total score > 100 are recommended to change jobs (Hang, 2013).

#### Statistical Analyses

SPSS 20.0 statistical software (SPSS, Inc, Chicago, IL, USA) was used for data analysis. Quantitative data are presented as means ± standard deviations, and qualitative data are presented as percentage. Differences in MBI and DASS-21 between pairs of groups were tested using one-way analysis variance (ANOVA) with *post-hoc* Bonferroni. DASS-21 and MBI results between the pre- and post-online psychological intervention periods were assessed using two-tailed Student's paired *t-*test. A two-tailed *p* < 0.05 was considered statistically significant.

## Results

Regarding the specialty of the 210 participants included, the proportion of internal medicine students was 18.57%, the proportion of surgical students was 20.48%, the proportion of anesthesia students was 23.81%, the proportion of pediatrics students was 11.43%, and 25.71% of them were in other majors. In total, there are 33.81% male participants, and 66.19% female participants, and 60.48% of them were aged between 20 and 25 years. Among the medical residents who were assessed, 27.14% were in their first year, 35.72% were in their second year, and 37.14 were in their third year. Table 1 shows that there was no significant difference in terms of gender, birthplace, age, and grade among the medical residents of different majors (*P* > 0.05, respectively). There were significant differences in the working environment and communication time with colleagues between medical residents from different majors (*P* < 0.05, respectively). Particularly, 28% of anesthesia residents reported that they felt very depressed about their working environment, which was significant higher than that of residents from other different majors. Only 16% of anesthesia residents stated that their working environment was relaxing; however, 53.85% of internal medicine residents indicated a relaxing environment. Furthermore, the proportion of anesthesia residents who rarely communicated with their colleagues daily was significantly higher than residents from different majors. And 79.07% of operation department students had more than 4 night shifts per month, which was higher than that among other majors. Regarding the health condition among medical students, although a majority of students reported to be healthy, the proportion of students who reported to have poor health was higher than those who reported to have average health.

**Table 1:** The demographics among different majors.

	**Total** ***n =* 210**	**Anesthesia**	**Internal medicine**	**OD**	**Pediatrics**	**Other majors**	
**Characteristics**	**(n) %**	**(n) %**	**(n) %**	**(n) %**	**(n) %**	**(n) %**	***P-*value**
**Gender**							0.629
Male	71	10	8	8	8	10	
	33.81%	20.00%	20.51%	18.60%	33.33%	18.52%	
Female	139	40	31	35	16	44	
	66.19%	80.00%	79.49%	81.39%	66.67%	81.48%	
**Birth place**							0.681
Countryside	101	22	18	23	12	21	
	48.10%	44.00%	46.15%	53.49%	50.00%	38.89%	
City	109	28	21	20	12	33	
	51.90%	56.00%	53.85%	46.51%	50.00%	61.11%	
**Age**							0.769
<20 years old	0	0	0	0	0	0	
	0	0	0	0	0	0	
20–25 years old	127	30	27	26	14	30	
	60.48%	60.00%	69.23%	60.47%	58.33%	55.56%	
25–30 years old	76	18	11	17	9	21	
	36.19%	36.00%	28.21%	39.53%	37.50%	38.89%	
≥30 years old	7	2	1	0	1	3	
	3.33%	4.00%	2.56%	0	4.17%	5.55%	
**Grade**							0.849
First year	57	19	15	17	9	14	
	27.14%	38.00%	38.46%	39.53%	37.50%	25.93%	
Second year	75	16	14	16	6	23	
	35.72%	32.00%	35.90%	37.21%	25.00%	42.59%	
Third year	78	15	10	10	9	17	
	37.14%	30.00%	25.64%	23.26%	37.50%	31.48%	
**Communication time**							0.029
Basically no	66	28	6	12	6	14	
	31.43%	56%	15.38%	27.91%	25%	25.93%	
Occasionally	66	10	21	11	3	17	
	31.43%	20%	53.85%	25.58%	12.5%	31.48%	
Frequently	63	10	10	18	10	15	
	30%	20%	25.64%	41.86%	41.67%	27.78%	
Every day	19	2	2	2	5	8	
	9.05%	4%	5.13%	4.65%	20.83%	14.81%	
**Working environment**							0.022
Very depressed	33	14	3	6	7	3	
	15.71%	28%	7.69%	13.95%	29.17%	5.56%	
Depressed	56	20	10	10	2	14	
	26.67%	40%	25.64%	23.26%	8.33%	25.93%	
Common	72	8	5	19	12	28	
	34.26%	16%	12.82%	44.19%	50%	51.85%	
Relaxing	49	8	21	8	3	9	
	23.33%	16%	53.85%	18.60%	12.5%	16.67%	
**Health condition**							0.318
Healthy	96	24	20	18	12	22	
	45.71 %	48%	51.28%	41.86%	50%	40.74%	
Common	5	10	13	13	6	11	
	25.24%	20%	33.33%	30.23%	25%	20.37%	
Poor	61	16	6	12	6	21	
	29.05%	32%	15.38%	27.91%	25%	38.89%	
**Number of night shifts per month**							<0.001
0	17	2	3	0	2	10	
	8.10%	40.0%	7.69%	0	8.33%	18.52%	
1–2	30	14	1	2	0	13	
	14.29%	28.00%	2.56%	4.65%	0	24.07%	
3–4	61	28	11	7	6	9	
	29.05%	56.00%	28.21%	16.28%	25.00%	16.67%	
>4	102	6	24	34	16	22	
	48.57%	12.00%	61.54%	79.07%	66.67%	40.74%	

*OD, Operation Department*.

### Prevalence of Burnout Syndrome Among Medical Residents

As shown in Table 2, burnout scores were categorized in terms of the EE, DP, and PA. Regarding sub-scales of MBI, 26.67% of the study participants scored high on the EE sub-scale, 15.24% of them scored high on the DP sub-scale, and 25.71% had a low score on the PA sub-scale. There were significant differences in the EE, DP, and PA among the groups with different majors (*P* < 0.05, respectively). Based on this categorization, anesthesia residents were classified as having a lower PA score (17.30 ± 5.2), and higher EE (34.22 ± 8.5) and DP (16.54 ± 5.1) scores compared to residents from other majors. Pediatric residents reported the second lowest PA score (18.46 ± 6.7) and second highest EE (31.13 ± 6.9) and DP (13.83 ± 6.5) scores among medical residents from other majors.

**Table 2:** The comparison of MBI among different majors.

	**Emotional exhaustion**	**Depersonalization**	**Personal accomplishment**
Anesthesia	34.22 ± 8.5	16.54 ± 5.1	17.30 ± 5.2
OD	29.14 ± 10.3	11.93 ± 7.1	19.30 ± 5.7
Internal medicine	30.59 ± 9.4	12.79 ± 5.6	21.41 ± 5.6
Pediatrics	31.13 ± 6.9	13.83 ± 6.5	18.46 ± 6.7
Other majors	26.65 ± 9.6	12.65 ± 6.1	22.04 ± 4.1
F	4.620	4.309	6.521
P	0.001	0.002	<0.001

*MBI, Maslach Burnout Inventory; OD, Operation Department*.

### Comparison of the Mean Scores of Stress, Anxiety, and Depression of Medical Residents

As shown in Table 3, with respect to the three emotional states of DASS-21, there were statistically differences in the anxiety, depression, and stress score among the groups with different majors (*P* < 0.05, respectively). Anesthesia residents and pediatric residents reported high stress (17.92 ± 3.5 and 16.42 ± 3.8), anxiety (11.42 ± 3.4 and 11.00± 4.0), and depression scores (14.38 ± 3.1 and 12.71 ± 4.4). The score for each of the three emotional states in anesthesia residents was higher than that in pediatric residents (*P* < 0.05, respectively). Further, internal medicine residents had the lowest score for the three emotional states.

**Table 3:** The comparison of each emotional state of DASS among different majors.

	**Depression**	**Anxiety**	**Stress**
Anesthesia	14.38 ± 3.1	11.42 ± 3.4	17.92 ± 3.5
OD	12.23 ± 3.9	9.07 ± 4.1	14.98 ± 5.6
Internal medicine	10.10 ± 4.1	7.41 ± 3.1	14.08 ± 6.5
Pediatrics	12.71 ± 4.4	11.00 ± 4.0	16.42 ± 3.8
Other majors	10.00 ± 4.2	8.04 ± 4.4	14.26 ± 6.8
F	10.540	8.793	3.953
P	<0.001	<0.001	0.004

*DASS, the Depression Anxiety Stress Scale; OD, Operation Department*.

### Effect of Online Psychological Intervention on Stress/Anxiety/Depression of Medical Residents

**Figure 2:**
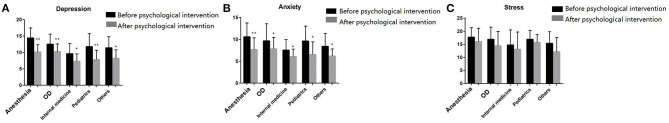
The comparision of DASS before and after psychological intervention among medical students from different majors. The DASS scale contains three emotional states of depression, anxiety, and stress. **(A)** Depression of DASS; **(B)** Anxiety of DASS; **(C)** Stress of DASS. *P < 0.05, **P < 0.001. OD, Operation Department.

Among the medical residents with a total MBI score between 50 and 75 who underwent online psychological intervention, the level of anxiety and depression after the online psychological intervention were significantly lower compared to pre-intervention (*P* < 0.05, respectively; Figure 2, Table 4). For anesthesia students, the levels of depression and anxiety pre- vs. post- online psychological intervention were 14.40 ± 3.1 vs. 10.13 ± 2.2 and 10.60 ± 3.2 vs. 7.67 ± 2.7, respectively. For operation department students, the levels of depression and anxiety pre- vs. post-online psychological intervention were 12.50 ± 3.1 vs. 10.25 ± 2.3 and 9.67 ± 3.9 vs. 7.83 ± 2.6, respectively. For internal medicine students, the levels of depression and anxiety pre- vs. post-online psychological intervention were 9.62 ± 3.1 vs. 7.31 ± 2.3 and 7.54 ± 2.4 vs. 6.08 ± 1.7, respectively. For pediatrics students, the levels of depression and anxiety pre- vs. post-online psychological intervention were 11.77 ± 4.0 vs. 7.85 ± 2.8 and 9.62 ± 3.4 vs. 6.54 ± 2.9, respectively. For students of other majors, the levels of depression and anxiety pre- vs. post-online psychological intervention were 11.40 ± 3.4 vs. 8.20 ± 2.6 and 8.40 ± 3.0 vs. 6.20 ± 1.6, respectively.

**Table 4:** The comparison of DASS before and after psychological intervention among medical students from different majors.

	**Depression**		**Anxiety**		**Stress**	
	**Before**	**After**	**Before**	**After**	**Before**	**After**
Anesthesia	14.40 ± 3.1	10.13 ± 2.2^**^	10.60 ± 3.2	7.67 ± 2.7^**^	17.73 ± 3.6	16.07 ± 5.1
OD	12.50 ± 3.1	10.25 ± 2.3^**^	9.67 ± 3.9	7.83 ± 2.6^*^	16.92 ± 4.7	14.42 ± 5.5
Internal medicine	9.62 ± 3.1	7.31 ± 2.3^*^	7.54 ± 2.4	6.08 ± 1.7^*^	14.77 ± 5.7	13.15 ± 6.6
Pediatrics	11.77 ± 4.0	7.85 ± 2.8^**^	9.62 ± 3.4	6.54 ± 2.9^*^	16.92 ± 3.4	15.77 ± 2.9
Other majors	11.40 ± 3.4	8.20 ± 2.6^*^	8.40 ± 3.0	6.20 ± 1.6^*^	15.40 ± 4.4	12.20 ± 5.4
Cohen's d	1.01		0.81		0.40	

*DASS, Depression, anxiety, and stress; OD, Operation Department*.

* *vs. DASS score before psychological intervention: P < 0.05;*

** *vs. DASS score before psychological intervention: P < 0.001*.

There were no differences in the level of stress pre- and post-online psychological intervention among the different majors (*P* > 0.05, respectively; Figure 2, Table 4). For anesthesia students, the level of stress pre- vs. post-online psychological intervention was 17.73 ± 3.6 vs. 16.07 ± 5.1. For operation department students, the level of stress pre- vs. post-online psychological intervention was 16.92 ± 4.7 vs. 14.42 ± 5.5. For internal medicine students, the level of stress pre- vs. post-online psychological intervention was 14.77 ± 5.7 vs. 13.15 ± 6.6. For pediatrics students, the level of stress pre- vs. post-online psychological intervention was 16.92 ± 3.4 vs. 15.77 ± 2.9. For students of other majors, the level of stress pre- and post-online psychological intervention was 15.40 ± 4.4 vs. 12.20 ± 5.4.

### Effect of Online Psychological Intervention on Burnout of Medical Residents

**Figure 3:**
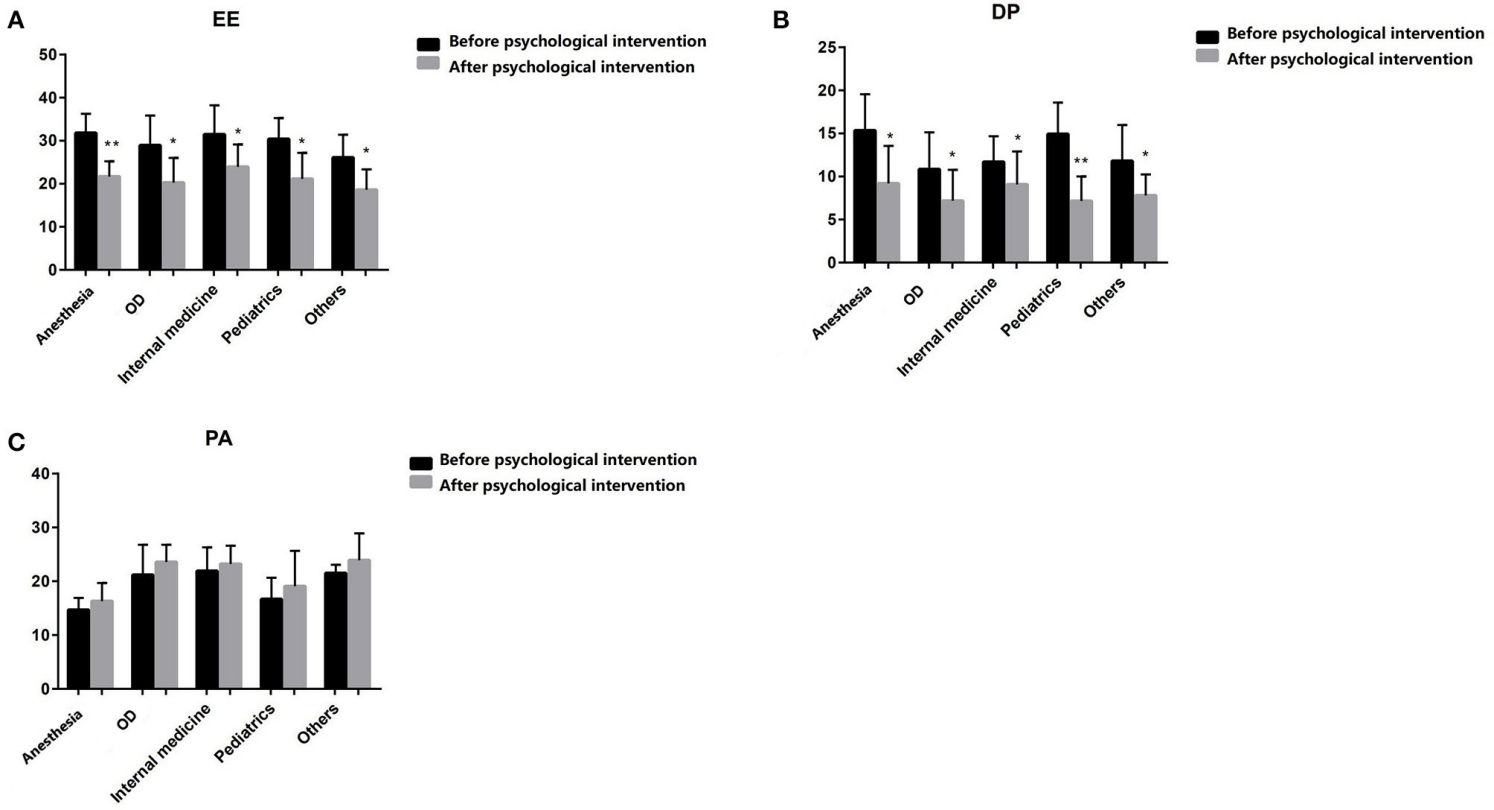
The comparision of MBI before and after psychological intervention among medical students from different majors. The MBI-Human Services version consists of 22 items with three subscales: emotional exhaustion (EE), depersonalization (DP), and personal accomplishment (PA). **(A)** Emotional exhaustion (EE) of MBI; **(B)** Depersonalization (DP) of MBI; **(C)** Personal accomplishment (PA) of MBI. *P < 0.05, **P < 0.001. OD, Operation Department.

The EE and DP scores were significantly decreased post-online psychological intervention compared to pre-online psychological intervention among medical residents with different majors (*P* < 0.05, respectively; Figure 3, Table 5). For anesthesia students, the levels of EE and DP pre- vs. post-online psychological intervention were 31.80 ± 4.5 vs. 21.67 ± 3.6 and 15.33 ± 4.2 vs. 9.20 ± 4.3, respectively. For operation department students, the levels of EE and DP pre- vs. post-online psychological intervention were 28.92 ± 6.9 vs. 20.25 ± 5.8 and 10.83 ± 4.3 vs. 7.17 ± 3.6, respectively. For internal medicine students, the levels of EE and DP pre- vs. post-online psychological intervention were 31.46 ± 6.8 vs. 23.92 ± 5.2 and 11.69 ± 3.0 vs. 9.08 ± 3.8, respectively. For pediatrics students, the levels of EE and DP pre- vs. post-online psychological intervention were 30.38 ± 4.9 vs. 21.15 ± 6.0 and 14.92 ± 3.7 vs. 7.15 ± 2.9, respectively. For students of other majors, the levels of EE and DP pre- vs. post-online psychological intervention were 26.10 ± 5.3 vs. 18.60 ± 4.7 and 11.80 ± 4.2 vs. 7.80 ± 2.4, respectively. There was no statistical difference in the PA score pre- and post-online psychological intervention among the different majors (*P* > 0.05, respectively; Figure 3, Table 5). For anesthesia students, the PA score pre- vs. post-online psychological intervention was 14.67 ± 2.3 vs. 14.40 ± 3.8. For operation department students, the PA score pre- vs. post-online psychological intervention was 21.17 ± 5.6 vs. 20.50 ± 5.5. For internal medicine students, the PA score pre- vs. post-online psychological intervention was 21.92 ± 4.4 vs. 22.23 ± 3.3. For pediatrics students, the PA score pre- vs. post-online psychological intervention was 16.69 ± 3.9 vs. 16.31 ± 5.1. For students of other majors, the PA score pre- vs. post-online psychological intervention was 21.50 ± 1.6 vs. 23.10 ± 3.0.

**Table 5:** The comparison of MBI before and after psychological intervention among medical students from different majors.

	**EE**		**DP**		**PA**	
	**Before**	**After**	**Before**	**After**	**Before**	**After**
Anesthesia	31.80 ± 4.5	21.67 ± 3.6^**^	15.33 ± 4.2	9.2 ± 4.3^*^	14.67 ± 2.3	14.40 ± 3.8
OD	28.92 ± 6.9	20.25 ± 5.8^*^	10.83 ± 4.3	7.17 ± 3.6^*^	21.17 ± 5.6	20.50 ± 5.5
Internal medicine	31.46 ± 6.8	23.92 ± 5.2^*^	11.69 ± 3.0	9.08 ± 3.8^*^	21.92 ± 4.4	22.23 ± 3.3
Pediatrics	30.38 ± 4.9	21.15 ± 6.0^*^	14.92 ± 3.7	7.15 ± 2.9^**^	16.69 ± 3.9	16.31 ± 5.1
Other majors	26.10 ± 5.3	18.60 ± 4.7^*^	11.80 ± 4.2	7.80 ± 2.4^*^	21.50 ± 1.6	23.10 ± 3.0
Cohen's d	1.57		1.26		−0.01	

*MBI, Maslach Burnout Inventory; OD, Operation Department; EE, emotional exhaustion; DP, depersonalization; PA, personal accomplishment;*

* *vs. MBI score before psychological intervention: P < 0.05;*

** *vs. MBI score before psychological intervention: P <0.001*.

## Discussion

This study assessed the prevalence of job burnout and the effect of online psychological intervention on the stress, anxiety, and depression among medical residents registered for post-graduate studies at Shengjing Hospital of China Medical University. The workload, lack of support, and loss of control at times undoubtedly contribute to a sense of emotional exhaustion in medical residents, and some may have had thoughts of “I just can't take it anymore.” Feelings of reduced personal accomplishment, being overworked, and emotional commitment to work can lead to depersonalization. These are the key elements of burnout. In our study, 87.62% of the medical residents from different majors reported to have either high or moderate emotional exhaustion. Moreover, 70.48% of the medical residents showed a high or moderate level of depersonalization, and 25.71% of the medical residents indicated a low sense of achievement. Shanafelt et al. reported a similar percentage (76%) of burnout among internal medicine residents in a USA residency program (Shanafelt et al., 2002). This is worrying since burned-out residents were reported to be two to three times more likely to report suboptimal patient care practices (Lu et al., 2015). Studies also suggest that medical resident burnout is related to deterioration of the general health status, substance abuse, anxiety, depression, suicidal thoughts, poor performance, and medical errors (Trockel et al., 2018; Brunsberg et al., 2019). The present study revealed that more than 49.05% of our respondents experienced a moderate to high level of anxiety, 79.52% of medical residents reported a moderate to high prevalence of depressive symptoms, and more than 82.86% of them experienced moderate to high stress levels. This complies with a previous study in Germany that included 435 doctors from six different disciplines found that burnout and depression were frequent occurrences in trainee doctors (Bernburg et al., 2015). Among all the medical residents assessed in our study, anesthesia residents reported the highest DASS scores than residents from other majors. The score for personal achievement was the lowest and emotional exhaustion was highest in anesthesia residents compared to residents from other different majors. Furthermore, there were 28% of anesthesia residents who reported that they felt very depressed about the working environment. And 56% of anesthesia residents indicated that they rarely communicate with their colleagues during daily work, which was significantly higher than residents from other different majors. The probable reasons may be that critical decision-making in a limited time and stressful situations is common in anesthesia practice, and anesthesia students need to be continuously vigilant to the monitoring of patients in operating rooms, which finally leads to severe mental fatigue. Further, training for anesthesia residents is unique when compared to other medical and surgical residents because they rarely work with peers. This structure decreases their peer-to-peer interactions and opportunities to build relationships for peer support. Moreover, our study found that following anesthesia residents, pediatric residents had the second highest score for each DASS emotional state. And 28% of pediatricians felt their working environment was particularly depressing. The results observed in pediatric residents may be due to the following reasons: firstly, the number of pediatricians in China is small: because most pediatricians considered critically high work stress, which was indicated by a substantial imbalance between effort and rewards received. The fewer pediatricians, the more they will experience more work stress, which will form a vicious circle (Weigl et al., 2015); secondly, the doctor-patient relationship is worse: as a special group of children, it is easier for doctors and parents to devote more affection. And many pediatric residents cannot handle compassion and the feelings of their children's parents well (Yao, 2020); finally, China has liberalized the two-child policy. This has led to an increasing trend in the number of Chinese children within a certain period of time, and has increased the work stress of pediatricians (Ye et al., 2019). In contrast to anesthesia and pediatric residents, 53.85% of internal medicine residents indicated having a relaxing environment, which was also consistent with the lowest emotional state scores observed in this group among different majors. A previous study demonstrated that long-term high psychological stress in young individuals may lead to high blood pressure, higher body mass index, higher cortisol levels, suppressed immune function, and decreased sleep quality (Starr et al., 2013). Therefore, it is necessary to implement relevant psychological interventions among these young medical residents through lifestyle modifications and by offering social support to the residents.

Previous studies proved that medical students who received interventions such as relaxation therapy, psychoeducation, and systematic desensitization for anxiety had lower scores post-assessment compared with students who did not receive any intervention (Kim and Kim, 2018; Newby et al., 2018; Manansingh et al., 2019). Compared with face-to-face interventions, Internet-based interventions are more accessible and affordable, and may meet the needs of promoting mental health interventions and preventing mental health issues. The use of mindfulness stress reduction interventions has been shown to improve attention, and related studies have also shown improvements in well-being and resilience (Bower et al., 2015). In our study, a total of 142 medical residents whose MBI score between 50 and 75 received the 3-months online psychological intervention, and only 63 of them finally completed the intervention. The possible reason for the low completion rate may be busy work arrangements, causing them not to have enough time to complete the online psychological intervention. Furthermore, some medical residents are unwilling to admit that they have psychological problems and are unwilling to disclose their psychological problems to others. The result of the online psychological intervention showed that it could effectively ameliorate EE and DP and alleviate the level of anxiety and depression among medical residents from different majors. Additionally, the degree of reduction was higher among anesthesia and pediatric residents when compared to the other majors. The possible reasons may be due to that internet-based interventions provide an alternative to face-to-face therapy, which is anonymous, self-paced, and easily accessible. Anesthesia and pediatric residents may be better release their pressure and enhance mental health through this method. However, this intervention had no statistically significant effects on the medical residents' sense of low personal achievement and stress level. The possible explanation for this phenomenon is that medical residents might experience pressure due to many factors, such as workforce demands, susceptibility to the uncertainties, and lack of control (Kuhn and Flanagan, 2017; Papaefstathiou et al., 2019). Medical residents may gain a sense of accomplishment as they master various of operations and become more proficient. Therefore, an online psychological intervention of only 3 months may not diminish the reduced personal sense of achievement or impact their stress level.

This study had several limitations. First, due to limited human and financial resources, we could not conduct follow-up research at this stage. Further, this study lacked a control group. Although this intervention showed significant positive effects on alleviating burnout in medical residents and emotional states over 3 months, a longitudinal study is required to explore the long-term influence of psychological intervention. Additionally, we only collected data from medical residents of Shengjing Hospital, so the results do not represent the Chinese medical resident population. Moreover, although our response rates were high, there may still be response bias. Medical residents with burnout might be more or less likely to respond to the survey.

## Conclusion

In conclusion, our findings reported a high rate of moderate to severe burnout in medical residents, especially anesthesia and pediatric residents, which indicated that it was necessary to focus on the implications of burnout on medical staff to reduce the incidence of suicide or medical errors. The online psychological intervention in our study proved that it not only had positive impacts on emotional exhaustion and depersonalization but also relieved psychological problems such as anxiety and depression in medical residents. Further, our study recommends anesthesia and pediatric residents should receive more psychological consultation and guidance and adopt a healthier lifestyle to prevent and manage stress-related problems.

## Data Availability Statement

The original contributions presented in the study are included in the article/supplementary material, further inquiries can be directed to the corresponding author/s.

## Ethics Statement

The studies involving human participants were reviewed and approved by Shengjing Hospital of China Medical University. The patients/participants provided their written informed consent to participate in this study.

## Author Contributions

JW, BS, YS, and JZ provided the conception or design of the work, drafted the work and revised it critically for important intellectual content, gave the final approval of the version to be published, agreed to be accountable for all aspects of the work in ensuring that questions related to the accuracy or integrity of any part of the work are appropriately investigated and resolved. YS also provided professional psychological intervention for our study.

## Conflict of Interest

The authors declare that the research was conducted in the absence of any commercial or financial relationships that could be construed as a potential conflict of interest.
